# P-1553. Impact of Urinary Tract Infection due to Carbapenem Resistant Gram Negative Bacteria in Patients with Kidney Transplant

**DOI:** 10.1093/ofid/ofae631.1720

**Published:** 2025-01-29

**Authors:** Giulia Soska Baldissera, Vinicius da Silva Gregory, Maria Helena da Silva Pitombeira

**Affiliations:** Federal University of Rio Grande do Sul, Porto Alegre, Rio Grande do Sul, Brazil; Federal University of Rio Grande do Sul, Porto Alegre, Rio Grande do Sul, Brazil; Federal University of Rio Grande do Sul, Porto Alegre, Rio Grande do Sul, Brazil

## Abstract

**Background:**

Carbapenem resistance is a fearsome reality, especially in transplant patients, and urinary tract infections may cause severe illness and graft loss.

**Methods:**

In this 10-year retrospective cohort we analyzed kidney transplanted patients with gram-negative carbapenem-resistant urinary tract infection (UTI). We evaluated overall prevalence, risk factors for 14-day clinical response and development of acute kidney injury or graft loss during infection. Patients ≥ 18 years old with symptomatic infections or loss of renal function attributed to UTI were included. We excluded patients with kidney abscesses or intra-abdominal complications, death within 24 h, non-active antimicrobial treatment.

**Results:**

From urocultures collected from 2013 – 2023, 11.583 isolated gram-negative bacteria, of which 14.2% were carbapenem resistant. From these, 87 patients were included. 72.4% were male, aged 56.9 ± 13.6 years old and median transplant time was 15.8 (2 - 89.6) months; 83.9% had upper UTI. The most prevalent bacteria was *Klebsiella pneumoniae*, with 75 samples (86.2%). 41 (47.1%) were carbapenemase producers: 29.8% serino and 18.3% metallo-beta lactamases. Patients were treated with: amikacin (58.6%) polymyxin B (47.1%), polymyxin E (32.2%), tigecycline (5.7%) ceftazidime-avibactam (8.0%). In 46.0% of the cases, these drugs were combined. 82.8% had complete resolution of symptoms and negative follow-up urine cultures in 14 days. Independent risk factors for non-response were baseline hemodynamic instability (p< 0.01) and polymyxin B treatment (p< 0.01). Control urocultures were collected from 79 (90.8%); 15 (18.9%) had the same bacteria. Sixty-eight patients (79.1%) who were not in dialysis at baseline were evaluated for AKI,from which 45 (66.1%) developed AKI during therapy and 8 (11.7%) had renal graft loss. 12 (13.8%) patients died in 30-days.

**Conclusion:**

Carbapenem resistant bacteria are increasingly prevalent in renal transplant patients and associated to AKI and graft loss potentially due to the use of nephrotoxic drugs. Polymyxin B and hemodynamic instability were independent preditors non-clinical response in 14-days, which should be further evaluated due to the non-urinary concentration of this antibiotic.

**Disclosures:**

**All Authors**: No reported disclosures

